# Validating administratively derived frailty scores for use in Veterans Health Administration emergency departments

**DOI:** 10.1111/acem.14705

**Published:** 2023-03-28

**Authors:** Sharmistha Dev, Andrew A. Gonzalez, Jessica Coffing, James E. Slaven, Shantanu Dev, Stan Taylor, Carrie Ballard, S. Nicole Hastings, Dawn M. Bravata

**Affiliations:** ^1^ Center for Health Information and Communication Roudebush VA Medical Center Indianapolis Indiana USA; ^2^ Regenstrief Institute Indianapolis Indiana USA; ^3^ Department of Emergency Medicine Indiana University Indianapolis Indiana USA; ^4^ Department of Surgery Indiana University Indianapolis Indiana USA; ^5^ Department of Biostatistics Indiana University–Purdue University Indianapolis Indianapolis Indiana USA; ^6^ Department of Computer Science The Ohio State University Columbus Ohio USA; ^7^ Center of Innovation to Accelerate Discovery and Practice Transformation Durham VA Health Care System Durham North Carolina USA; ^8^ Department of Medicine Duke University School of Medicine Durham North Carolina USA; ^9^ Department of Medicine Indiana University Indianapolis Indiana USA

## Abstract

**Objectives:**

Frailty is a clinical syndrome characterized by decreased physiologic reserve that diminishes the ability to respond to stressors such as acute illness. Veterans Health Administration (VA) emergency departments (ED) are the primary venue of care for Veterans with acute illness and represent key sites for frailty recognition. As questionnaire‐based frailty instruments can be cumbersome to implement in the ED, we examined two administratively derived frailty scores for use among VA ED patients.

**Methods:**

This national retrospective cohort study included all VA ED visits (2017–2020). We evaluated two administratively derived scores: the Care Assessment Needs (CAN) score and the VA Frailty Index (VA‐FI). We categorized all ED visits across four frailty groups and examined associations with outcomes of 30‐day and 90‐day hospitalization and 30‐day, 90‐day, and 1‐year mortality. We used logistic regression to assess the model performance of the CAN score and the VA‐FI.

**Results:**

The cohort included 9,213,571 ED visits. With the CAN score, 28.7% of the cohort were classified as severely frail; by VA‐FI, 13.2% were severely frail. All outcome rates increased with progressive frailty (*p*‐values for all comparisons < 0.001). For example, for 1‐year mortality based on the CAN score frailty was determined as: robust, 1.4%; prefrail, 3.4%; moderately frail, 7.0%; and severely frail, 20.2%. Similarly, for 90‐day hospitalization based on VA‐FI, frailty was determined as prefrail, 8.3%; mildly frail, 15.3%; moderately frail, 29.5%; and severely frail, 55.4%. The c‐statistics for CAN score models were higher than for VA‐FI models across all outcomes (e.g., 1‐year mortality, 0.721 vs. 0.659).

**Conclusions:**

Frailty was common among VA ED patients. Increased frailty, whether measured by CAN score or VA‐FI, was strongly associated with hospitalization and mortality and both can be used in the ED to identify Veterans at high risk for adverse outcomes. Having an effective automatic score in VA EDs to identify frail Veterans may allow for better targeting of scarce resources.

## INTRODUCTION

Frailty is a clinical syndrome characterized by decreased physiologic reserve across multiple organ systems resulting in a diminished ability to respond to stressors such as acute illness.^1^, ^2^, ^3^ Frailty has been linked to multiple adverse patient‐centered outcomes.^4^ For example, frail patients who are admitted to the hospital have longer hospital lengths of stay, increased in‐hospital and long‐term mortality, increased risk of postoperative complications, and increased readmissions.^4^, ^5^, ^6^, ^7^ Similarly, frail patients are also more likely to have falls, functional disability, fractures, and poor quality of life.^8^, ^9^, ^10^ These increased risks of adverse outcomes provide a robust rationale for identifying frail individuals and intervening early.

Although age is not the only component of frailty, previous studies have shown a strong correlation between age and increased frailty. Gaining a deeper understanding of frailty has become ever more pressing as the population of the United States continues to age: 20% of the U.S. population is predicted to be older than 65 years by 2040.^11^ This demographic shift is also evident in the U.S. Veteran population with over half of Veterans projected to be older than 65 years in the next 30 years.^12^ Veterans who use the Veterans Health Administration (VA) when compared both to the general population and to Veterans who use non‐VA community resources are older and have more complex medical needs due to a higher prevalence of physical and mental health conditions.^13^ Within the VA, frailty affects three out of every 10 Veterans over the age of 65 years, which is three times higher than among non‐Veterans due to a combination of older age and increased burden of comorbid conditions.^14^, ^15^

The emergency department (ED) represents an important venue of care for older adults. From 2006 to 2015, the ED visit rate was highest for patients over the age of 65 years compared with all other age groups.^16^ Within the VA, this age group accounted for over 45% of all ED visits, which is more than twice the rate for the same age group in the civilian population.^17^, ^18^ Moreover, this age group is at an increased risk of adverse outcomes compared to the general population.^19^

Given the large proportion of ED visits by elderly patients and the association between age, frailty, and adverse post‐ED visit outcomes, assessing for frailty within the ED may lead to successful identification and improve our ability to address complex care issues associated with frail patients.

Although there are numerous disease‐specific screening tools in use in VA EDs, assessing for frailty in the ED is a relatively new concept.^20^, ^21^ The ED presents unique constraints for screening of patients. Using instruments that require patient questionnaires in the ED can be cumbersome and time‐intensive.^20^, ^22^ To ensure that patient care is not affected adversely, screening procedures in the ED must be brief. Screens performed in the ED for health‐related social needs, substance abuse disorders, or behavioral health problems have led to successful intervention to address identified patient problems.^23^, ^24^, ^25^ Previous studies of frailty scores performed in the ED found that individuals identified as severely frail had increased risk of repeat ED visits, admission, and mortality.^26^, ^27^, ^28^ To date there have been no published studies on the application of frailty scores within the VA ED population.

In this context, we sought to evaluate a frailty assessment using only data obtainable from the electronic health record, which can be automatically calculated. We chose to use the VA‐Frailty Index (VA‐FI) and the Care Assessments Needs (CAN) score as these scores do not require questionnaires, can be calculated automatically using administrative data, and hence can be used efficiently in the ED setting. We aimed to describe the prevalence of frailty in the VA ED population and examine its association with hospitalizations and mortality.

## METHODS

### Study design

This is a nationwide retrospective cohort study using observational data from the VA Corporation Data Warehouse (CDW). The CDW is a national repository of VA administrative claims and electronic health data. The data were accessed through the VA Informatics and Computing Infrastructure (VINCI).^29^ VINCI is a secure and virtual platform that provides software development tools for the purposes of querying and analyzing national VA data. This study was approved by the Richard L. Roudebush VA Medical Center Research and Development Committee and the Indiana University Institutional Review Board.

### Two frailty scores

To characterize frailty, we used two administratively derived scores that can be easily found or derived using the VA CDW data. The Care Assessment Needs (CAN) score is a predictive analytic tool that was developed within the VA to help primary care providers make management or care coordination decisions.^30^ The score was designed to predict risk of hospitalization or death at 90 days and 1 year. The score is not a frailty score; however, the methodology for calculation of the CAN score is consistent with the cumulative deficit model, similar to other frailty scores, and the CAN score has been validated against other frailty scores for possible use as a screening tool.^31^, ^32^ All VA patients who are assigned a primary care physician and have received at least one primary care service within the VA have a CAN score within the CDW data. The most recent score prior to the index ED visit was used for this study. To standardize the cohort and minimize time variance of CAN score, we limited to the most recent available score up to 1 year prior to the ED visit. The range of the CAN score is between 0 and 99. The score can be further divided into robust (<65), prefrail (≥65 to <85), moderately frail (≥85 to <95), and severely frail (≥95).^31^, ^33^

The VA‐FI was created in 2019 and is also based on the cumulative deficit model of frailty scores. It uses International Classification of Disease (ICD)‐9/10 codes and Current Procedural Terminology (CPT) codes and has been validated to predict risk of death for all VA patients older than 65 years.^15^, ^34^, ^35^ The VA‐FI is based on 31 diagnoses; a ratio is formulated with the presence of these diagnoses as the numerator and 31 as the denominator to produce a score between 0 and 1. The score can be further divided into prefrail (≤0.1), mildly frail (>0.1 to ≤0.2), moderately frail (>0.2 to ≤0.4), and severely frail (>0.4).^15^ For our study we calculated the VA‐FI at the beginning of each ED visit; we did not include the diagnoses of the ED visit in the calculation.

### Study population

We included all Veterans who had an ED visit at any VA ED within a VA medical center from January 1, 2017, to December 31, 2020. Urgent care visits were not included. Each ED visit was treated separately. Patients seen in VA EDs that cared for fewer than 10 patients annually were excluded. We also excluded patients who did not have a CAN score available or who did not have the data available for calculation of the VA‐FI.

### Outcome measures

Our outcomes of interests were hospitalizations and mortality. We defined a hospitalization as an inpatient stay after an index ED visit. We used 30‐day and 90‐day hospitalization rates. These time points were used because they have been previously validated with the CAN score and represent time points that may reflect care provided during the index ED visit.

For mortality we used 30‐day, 90‐day, and 1‐year mortality. Mortality was confirmed using the National Death Index, which is a national file compiled from computer files submitted by state vital statistics offices and includes identifying death record information.^36^

### Covariates

We extracted CDW data on age, gender, race, ethnicity, Charlson Comorbidity Index, and hospital complexity. Each VA hospital is classified into one of five levels of complexity with 1a as the most complex and three as the least complex. Complexity level is based on the patient population served, the complexity of the clinical services including acute care and the surgical specialties available, and involvement in resident and medical student education and research.^37^ The most complex hospitals (1a) have the largest number of available specialties and critical care beds. We also categorized hospitals as urban versus rural based upon the VA's internal methodology of categorizing VA facilities as such.^38^

### Statistical analysis

We analyzed differences in demographic characteristics across calendar years and frailty groups using analysis of variance models for continuous variables and chi‐square tests for categorical variables. To assess the predictive accuracy of both the CAN score and the VA‐FI, we performed logistic regression models using dichotomous versions (severely frail vs. not severely frail) of both the CAN score and the VA‐FI across my outcome variables. To compare the two scores, we calculated the c‐statistic across the models. We verified multicollinearity, independence, no severe outliers, sufficient large sample size, and a rough linear relationship between the scores and our outcome variables. All analyses were performed using SAS version 9.4 (SAS Institute).

## RESULTS

There were a total of 9,745,659 ED visits from 2017 to 2020 across 103 VA EDs of which we evaluated 9,213,571 ED visits (Appendix 1); 532,088 ED visits (5.4%) were excluded as they did not have available CAN scores. The number of ED visits were stable at around 2.4 million per year from 2017 to 2019. However, in 2020, there were almost 20% fewer ED visits, likely secondary to COVID‐19. Patients were commonly older than 50 years, male, White, and not Hispanic. While all comparisons of patient characteristics were statistically significant (*p* < 0.05) due to the large sample size, there were no clinically meaningful or policy relevant differences between groups.

Table 1 provides patient demographics of the ED visits across both the CAN score and the VA‐FI score across frailty groups. For the entire population, the most recent CAN score prior to an ED visit was a mean of 10.4 days, median of 4 days and standard deviation of 28.1 days prior to the ED visit. Both scores showed similar trends in terms of older age with progressive frailty. Similarly, the percentage of males, percentage of White, and percentage of those that were not Hispanic also increased with progressive frailty. When comparing the CAN score to the VA‐FI, the average age for the Robust group for the CAN score and the prefrail group for the VA‐FI was similar (52.1 vs. 51.7, respectively). However, for all other groups of frailty, the VA‐FI had a higher average age; for example, among severely frail the mean age was 74.1 years based on VA‐FI classification and 69.4 years based on CAN score classification. Those categorized as severely frail by VA‐FI and CAN scores had a similar percentage of Male and Hispanic ethnicity, but severely frail VA‐FI patients were more likely to be White than those classified as severely frail by the CAN score. Both VA‐FI and CAN scores had a wide range for Charlson Comorbidity Index with similar medians as there were a large number of patients with a Charlson Comorbidity Index of 0.

**TABLE 1:** Demographics stratified by scoring system and severity.

Demographics	CAN score				VA‐FI			
	Robust	Prefrail	Moderately frail	Severely frail	Prefrail	Mildly frail	Moderately frail	Severely frail
	<65	65–85	86–94	≥95	<0.1	0.1–<0.2	0.2–0.4	>0.4
Age (years), mean (±SD)	52.1 (±15.4)	60.1 (±14.8)	64.9 (±13.7)	69.4 (±13.0)	51.7 (±15.7)	61.5 (±14.1)	68.2 (±11.8)	74.1 (±10.4)
Gender, *n* (%)								
Female	319,533 (12.5)	274,239 (13.7)	201,594 (10.1)	139,750 (5.3)	432,458 (14.0)	264,419 (11.3)	185,420 (7.2)	52,819 (4.3)
Male	2,244,571 (87.5)	1,726,229 (86.3)	1,804,314 (90.0)	2,503,341 (94.7)	2,660,441 (86.0)	2,075,705 (88.7)	2,378,777 (92.8)	1,163,532 (95.7)
Race, *n* (%)								
White	1,630,181 (66.3)	1,283,573 (66.3)	1,332,231 (68.2)	1,793,109 (69.4)	1,919,466 (64.6)	1,503,791 (66.3)	1,739,362 (69.6)	876,475 (73.5)
Black	715,063 (29.1)	592,875 (30.6)	571,248 (29.3)	737,030 (28.5)	923,823 (31.1)	696,501 (30.7)	702,102 (28.1)	293,790 (24.6)
Pacific Islander	23,359 (1.0)	15,781 (22.6)	14,430 (0.7)	16,351 (0.6)	26,909 (0.9)	18,092 (0.8)	17,988 (0.7)	6932 (0.6)
Asian	37,596 (1.5)	16,524 (0.9)	11,205 (0.6)	10,613 (0.4)	40,409 (1.4)	18,451 (0.8)	12,736 (0.5)	4342 (0.4)
Unknown	51,829 (2.1)	26,704 (1.4)	23,375 (1.2)	27,850 (1.1)	59,803 (2.0)	30,577 (1.4)	28,134 (1.1)	11,244 (0.9)
Ethnicity, *n* (%)								
Hispanic	243,982 (9.8)	159,205 (8.2)	143,086 (7.3)	179,395 (7.0)	281,918 (9.4)	181,272 (8.0)	180,131 (7.2)	82,347 (6.9)
Not Hispanic	2,239,754 (90.2)	1,786,819 (91.8)	1,809,993 (92.7)	2,398,538 (93.0)	2,716,850 (90.6)	2,096,151 (92.0)	2,318,097 (92.8)	1,104,006 (93.1)
Smoking status, *n* (%)								
Current	643,575 (42.8)	589,235 (44.0)	653,433 (46.1)	997,687 (51.6)	954,005 (50.3)	750,573 (46.4)	815,084 (44.8)	364,268 (42.3)
Former	329,526 (21.9)	307,299 (22.9)	324,507 (22.9)	398,506 (20.6)	367,173 (19.3)	360,274 (22.3)	430,754 (23.7)	201,637 (23.4)
Never	529,900 (35.3)	443,200 (33.1)	440,317 (31.1)	537,744 (27.8)	577,496 (30.4)	505,590 (31.3)	572,481 (31.5)	295,594 (34.3)
Charlson Comorbidity Index, median (range)	0 (0–17)	0 (0–18)	0 (0–20)	0 (0–25)	0 (0–19)	0 (0–19)	0 (0–22)	0 (0–25)

Abbreviations: CAN, Care Assessment Needs; VA‐FI, VA‐Frailty Index.

To evaluate percentage of younger patients that compose higher frailty groups, we stratified the patients by age (greater or younger than 65 years old) across frailty groups. Of those categorized in the severely frail group, those less than 65 years old composed 32.8% by CAN score and 19.5% by VA‐FI. Of those categorized in the moderately frail group, those less than 65 years old composed 43.2% by CAN score and 35.1% by VA‐FI.

### Frailty prevalence

Table 2 provides the data for the outcomes across both CAN score and VA‐FI frailty strata. According to the CAN score classification, severely frail composed 28.7% of the entire VA ED population. In contrast, according to the VA‐FI, severely frail composed 13.2% of the entire VA ED population.

**TABLE 2:** Mortality and hospitalizations stratified by severity and scoring system.

Outcome	CAN score				VA‐FI			
	Robust	Prefrail	Moderately frail	Severely frail	Prefrail	Mildly frail	Moderately frail	Severely frail
	<65	65–85	86–94	≥95	<0.1	0.1–0.2	0.2–0.4	>0.4
Count	2,564,104 (27.8)	2,000,468 (21.7)	2,005,908 (21.8)	2,643,091 (28.7)	3,092,899 (33.6)	2,340,124 (25.4)	2,564,197 (27.8)	1,216,351 (13.2)
30‐day hospitalization	210,673 (8.2)	226,157 (11.3)	325,223 (16.2)	855,324 (32.4)	219,275 (7.1)	300,177 (12.8)	602,844 (23.5)	495,081 (40.7)
90‐day hospitalization	233,741 (9.1)	262,458 (13.1)	397,645 (19.8)	1,150,197 (43.6)	255,583 (8.3)	357,687 (15.3)	756,341 (29.5)	674,431 (55.4)
30‐day mortality	5998 (0.2)	10,793 (0.5)	21,078 (1.1)	89,748 (3.4)	8914 (0.3)	19,141 (0.8)	54,856 (2.1)	44,706 (3.7)
90‐day mortality	13,567 (0.5)	24,384 (1.2)	49,629 (2.5)	209,715 (7.9)	21,819 (0.7)	43,011 (1.8)	124,829 (4.9)	107,636 (8.9)
1‐year mortality	36,849 (1.4)	67,987 (3.4)	139,558 (7.0)	535,043 (20.2)	63,113 (2.0)	112,221 (4.8)	313,788 (12.2)	290,315 (23.9)

*Note*: Data are reported as *n* (%).

Abbreviations: CAN, Care Assessment Needs; VA‐FI, VA‐Frailty Index.

### Association between frailty severity, hospitalizations, and mortality

For both the CAN score and the VA‐FI, increasing frailty was associated with higher rates of 30‐day and 90‐day hospitalization as well as 30‐day, 90‐day, and 1‐year mortality. For example, the 90‐day hospitalization rate increased with progressive frailty based on VA‐FI: prefrail, 8.3%; mildly frail, 15.3%; moderately frail, 29.5%; and severely frail, 55.4%. In another example, 1‐year mortality increased with progressive frailty based on the CAN score: robust, 1.4%; prefrail, 3.4%; moderately frail, 7.0%; and severely frail, 20.2%. The largest increase in all outcomes measured using either the CAN score or the VA‐FI occurred from the moderately frail group to the severely frail group. The patients identified as severely frail by the VA‐FI score had higher rates of all of the outcomes compared with patients identified as severely frail by the CAN score (Table 2).

Table 3 provides detailed data for the severely frail patients identified by the CAN score. The highest percentage of severely frail patients by the CAN score had a score of 99 (34.7%). The largest absolute increases in all outcomes occurred from a CAN score of 98–99. At the highest CAN score of 99, the 90‐day hospitalization rate was 57.7% and the 1‐year mortality rate was 30.2%.

**TABLE 3:** Further stratification of severely frail patients by CAN score.

CAN score	Patients	30‐day hospitalization	90‐day hospitalization	30‐day mortality	90‐day mortality	1‐year mortality
95	317,444 (12.0)	68,872 (21.7)	87,465 (27.6)	5284 (1.7)	12,332 (3.9)	34,221 (10.8)
96	370,537 (14.0)	89,550 (24.2)	115,363 (31.2)	7158 (1.9)	16,774 (4.5)	46,686 (12.6)
97	450,475 (17.0)	123,550 (27.4)	161,823 (35.9)	10,514 (2.3)	24,691 (5.5)	67,355 (15.0)
98	586,777 (22.2)	191,030 (32.6)	256,521 (43.8)	17,578 (3.0)	41,749 (7.1)	109,693 (18.7)
99	917,858 (34.7)	382,322 (41.7)	529,025 (57.7)	49,214 (5.4)	114,169 (12.4)	277,088 (30.2)

*Note*: Data are reported as *n* (%).

Abbreviation: CAN, Care Assessment Needs.

Appendix 2 display frailty by facility characteristics. With regard to hospital complexity, as expected the largest number of ED patients were cared for within 1a facilities (50.9%). Among ED visits at 1a facilities, 13.8% and 29.3% were for severely frail patients by VA‐FI and CAN scores, respectively. Low‐complexity (3) facilities had the lowest percentage of severely frail patients among their total ED population, by both VA‐FI and CAN scores. Overall, there was heterogeneity across complexity of facilities in their mean VA‐FI, mean CAN score, and percentage of severely frail: the more complex a facility, the higher the mean VA‐FI and CAN score and the percentage of severely frail. Appendix 2 demonstrates that, as expected, the vast majority of ED patients were seen within urban facilities. Urban facilities had a slightly higher percentage of severely frail patients by both VA‐FI and CAN score. Patients in both urban and rural facilities had similar mean VA‐FI (0.21) and mean CAN scores (73).

**FIGURE 1:**
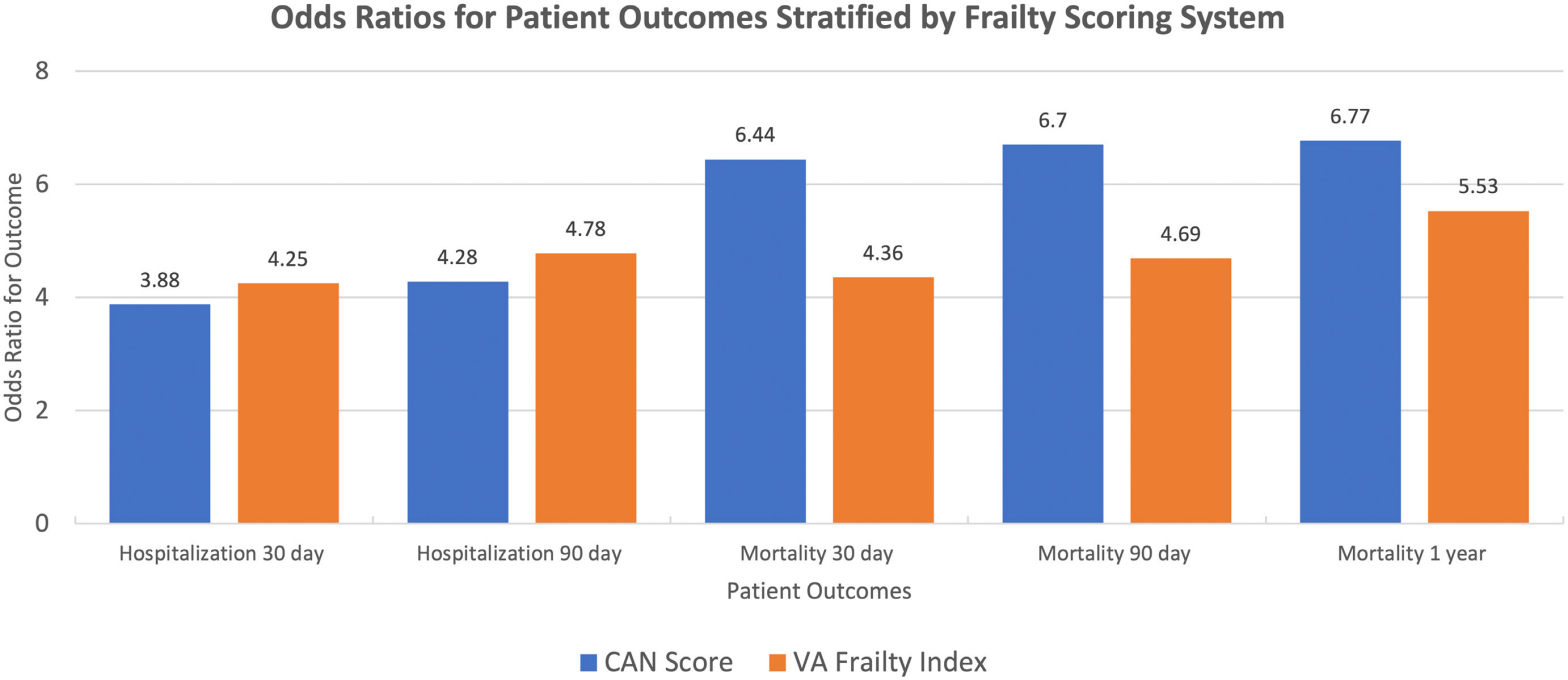
ORs for patient outcomes stratified by frailty scoring system. CAN score, Care Assessments Needs score.

Figure 1 displays the odds ratios (ORs) for all outcomes stratified by frailty score with frailty taken as a dichotomous variable of severely frail versus not severely frail. The VA‐FI had higher ORs for 30‐ and 90‐day hospitalizations, and the CAN score had higher ORs for 30‐day, 90‐day, and 1‐year mortality. The c‐statistics for the models examining the association between frailty classified by the CAN score and outcomes were higher than that for models using the VA‐FI to classify frailty (e.g., 1‐year mortality 0.721 vs. 0.659).

## DISCUSSION

Although frailty affects a significant proportion of the VA population, there is limited literature about the prevalence of frailty within the VA ED population. Our study sought to apply two administratively derived measures of frailty on the VA ED population and assess their association with hospitalizations and mortality. We confirmed that frailty is highly prevalent among the VA ED population. Specifically, we found that more than 40% of the total VA ED population was either moderately or severely frail using either the CAN score or the VA‐FI. Furthermore, we showed that although age is correlated with frailty, there is still a strong proportion of Veterans who are younger than 65 years old but are still considered moderately and severely frail. Increasing frailty, identified by both the CAN score and the VA‐FI, was strongly associated with hospitalization and mortality rates. Both scores successfully segregated patients into meaningfully different risk categories. For example, the risk of 90‐day hospitalization was only 8.3% (by VA‐FI) or 9.1% (by CAN) for robust or prefrail patients but was quite staggeringly, 44% (by CAN) or 55% (by VA‐FI) for severely frail patients. Similarly, patients were classified into distinct 1‐year mortality risk groups: from 1.4% (by CAN) or 2.0% (by VA‐FI) for robust or prefail patients to over 20% for severely frail VA ED patients. These results indicate that administratively derived frailty scores can be used to identify frailty among the VA ED population.

Having a frailty score that can predict hospitalization and mortality may allow for point‐of‐care identification and objective risk stratification of severely frail patients. Several studies have shown that applications of frailty scores can help target interventions to high‐risk patients. The ED is unique among health care venues in that it provides the rare opportunity to bring all the care coordination resources that the hospital has available to the patient within in a very short time course. For example, to improve care for elderly Veterans within VA EDs, the VA launched the geriatric ED initiative.^39^ The initiative utilizes social work and VA home resources to support physicians in preventing hospitalizations and improving quality of life. Another example of a resource that could be applied to the care of high‐risk, severely frail patients in the VA ED is access to clinical pharmacists for medication reconciliation and discontinuation of potentially inappropriate medications.

Although frailty scores have not been used to screen patients within the VA ED population, they have been applied in other settings within the VA. Hall et al.^40^ applied frailty scores as a preoperative screening for all patients undergoing major elective noncardiac surgery to direct perioperative planning. After application of this frailty screening, postoperative mortality was significantly reduced. Similarly, the CAN score has been widely available for Veterans receiving primary care within the VA and has been used to identify high‐risk patients within primary care and guide care coordination efforts for these patients.^41^

Administratively derived frailty scores, such as the CAN score and the VA‐FI, are an efficient method of assessing for frailty within the time‐limited ED setting. Frailty scores that require patient interview or physical assessment can be burdensome for the ED clinician to administer and could delay care for a patient's presenting ED complaint. Furthermore, unlike other frailty measures that can be disease or specialty specific like the Carolina Frailty Index or the Emergency Surgery Frailty Index (specific for emergency surgery patients), the CAN score and the VA‐FI are broadly applicable, therefore providing a source of common ground among specialties for patients requiring multidisciplinary care.^42^, ^43^

In terms of application to VA EDs, we envision using the CAN score and the VA‐FI in conjunction. The CAN score is readily available to all VA patients who receive primary care within the VA (which is the vast majority of the VA ED population). Therefore, it could be implemented as a screening tool to identify severely frail VA ED patients. As our results have shown, the VA‐FI identifies severely frail patients with a modestly higher risk of adverse outcomes than the CAN score; therefore, future studies could assess the application of the VA‐FI to target the highest risk patients for interventions. Interventions that have previously been implemented for frail patients have varied from increased home services support to implementing telemonitoring. In one study of elderly frail patients in Switzerland, increasing home visits and having targeted standardized follow‐up for frail patients helped to decreased overall unnecessary hospitalizations.^44^ Another study found that providing transitional care, which ranged from home nursing services to physical therapy to discharged hospitalized frail patients helped to reduce readmissions at 6 months.^45^ Telemonitoring has also been another tool used to target frail patients: one study found that this helped to reduce readmission in frail post–cardiac surgery patients.^46^ Future research should also evaluate methods and feasibility of implementing such interventions from the ED and its effect on reducing hospitalizations and mortality among frail patients.

## LIMITATIONS

The use of administrative data can have several limitations including coding, data validity, and data availability. However, our study found less than 5% missingness for all characteristics studied. Our study was conducted within the VA, which limits generalizability to non‐VA EDs. In general, the Veteran population has a higher percentage of older, White, and male patients when compared to the general ED population.^47^ However, a strength of our approach was the inclusion of all Veterans cared for in any VA ED, which did not limit the frailty assessment to only older patients. This approach is particularly relevant to the Veteran ED population, which is known to have a higher comorbidity burden than non‐VA ED populations given that frailty can be based on older age with limited comorbidity or younger age with considerable comorbidity. Both frailty indexes were based on the cumulative deficit model and did not include laboratory or clinical data such as vital signs. However, this may be a strength as many of these variables can be missing from electronic health records.

## CONCLUSIONS

We demonstrated that both the VA‐Frailty Index and the Care Assessments Needs scores can be applied to Veterans Health Administration ED patients to identify frail Veterans at high risk of hospitalization and death. Future research should examine the use of these scores at the point of care to potentially improve outcomes for the most frail, high‐risk patients.

## AUTHOR CONTRIBUTIONS

Sharmistha Dev—study concept and design, acquisition of the data, analysis and interpretation of the data, drafting of manuscript, critical revision of manuscript, acquisition of funding. Andrew A. Gonzalez—study concept and design, analysis and interpretation of the data, critical revision of manuscript. Jessica Coffing—acquisition of the data. James E. Slaven—analysis and interpretation of the data, statistical expertise. Shantanu Dev—analysis and interpretation of the data, statistical expertise. Stan Taylor—analysis of the data. Carrie Ballard—study design, critical revision of the manuscript. S. Nicole Hastings—critical revision of the manuscript. Dawn M. Bravata—analysis and interpretation of the data, critical revision of manuscript

## FUNDING INFORMATION

This research was funded by Indiana Clinical Translational and Science Institute's Research Enhancement Award Program.

## CONFLICT OF INTEREST STATEMENT

The authors declare no conflicts of interest.

## Supporting information


Data S1.

## APPENDIX 1

### Overall demographics by year

**Table:**

	Overall	2017	2018	2019	2020
Total	9,213,571	2,367,971	2,419,710	2,449,736	1,976,154
Age					
Mean years (SD)	61.6 (15.8)	61.3 (15.7)	61.5 (15.8)	61.6 (15.8)	61.9 (15.7)
Median (range)	64 (19–110)	64 (19–107)	64 (19–109)	64 (19–110)	64
Interquartile range	52–72	52–71	52–72	52–72	52–73
Male gender, *n* (%)	8,278,455 (89.9)	2,136,547 (90.2)	2,174,373 (89.9)	2,197,353 (89.7)	1,770,182 (89.6)
Race, *n* (%)					
White	6,039,094 (65.6)	1,579,701 (66.7)	1,596,440 (66.0)	1,594,941 (65.1)	1,268,012 (64.2)
Black or African American	2,616,216 (29.3)	651,858 (27.5)	680,572 (28.1)	703,806 (28.7)	579,980 (29.3)
Pacific Islander	69,921 (0.8)	17,201 (0.7)	18,319 (0.8)	18,731 (0.8)	15,670 (0.8)
Asian	75,938 (0.8)	18,313 (0.8)	19,549 (0.8)	20,636 (0.8)	17,440 (0.9)
Unknown	129,758 (1.4)	31,095 (1.3)	31,969 (1.3)	35,559 (1.5)	31,135 (1.6)
Missing	282,644 (3.1)	69,803 (2.9)	72,861 (3.0)	76,063 (3.1)	63,917 (3.2)
Ethnicity, *n* (%)					
Hispanic	725,668 (7.9)	180,965 (7.6)	189,007 (7.8)	194,818 (8.0)	160,808 (8.1)
Not Hispanic	8,235,104 (89.4)	2,122,797 (89.6)	2,165,803 (89.5)	2,186,961 (89.3)	1,759,543 (89.0)
Missing	252,799 (2.7)	64,209 (2.7)	64,830 (2.7)	67,957 (2.8)	55,803 (2.8)
Charlson Co‐morbidity Index					
Median (range)	0 (0–25)	0 (0–24)	0 (0–24)	0 (0–25)	0 (0–22)
Mean	0.468	0.248	0.350	0.465	0.832

## APPENDIX 2

### Percentage of severely frail patients by hospital characteristics

**Table:**

Facility characteristics	Number of patients (% of total)	Mean age	Mean VA‐FI	% Severely frail by VA‐FI (>0.4)	Mean CAN score	% Severely frail by CAN (≥95)
Complexity						
1a	4,585,418 (50.9)	60.9 (15.9)	0.21	13.8	73	29.3
1b	2,171,619 (24.1)	61.7 (15.5)	0.21	13.4	73	28.4
1c	1,456,848 (16.2)	62.6 (15.6)	0.21	12.9	74	29.3
2	587,865 (6.5)	63.0 (15.8)	0.20	11.0	73	26.5
3	204,120 (2.3)	64.6 (15.6)	0.19	9.5	71	23.5
Urban vs. Rural						
Urban	8,890,048 (96.5)	61.5 (15.8)	0.21	13.3	73	28.8
Rural	323,517 (3.5)	64.5 (15.4)	0.21	11.7	73	26.6
