# Supplementary material for: Validating administratively derived frailty scores for use in Veterans Health Administration emergency departments
Source: Acad Emerg Med. 2023 Mar 28;30(4):349–58. doi: 10.1111/acem.14705 (PMC10162447; doi:10.1111/acem.14705)
Supplement: Supplementary file 1 — Data S1. [file ACEM-30-349-s001.zip › acem14705-sup-0003-AppendixS3.docx]

**Appendix 3: Care Assessment Need Score Data Elements**

Sourced from:

Ruiz JG, Priyadarshni S, Rahaman Z, et al. Validation of an automatically generated screening score for frailty: The care assessment need (CAN) score. BMC Geriatr 2018; 10.1186/s12877-018-0802-7

1. Demographics:

Age (≥65)

Marital status

Service connection more than 50%*

Sex

1. Chronic Illness:

Atrial fibrillation

Atherosclerotic Peripheral Vascular Disease.

COPD

Dementia

Depression

Deyo-Charlson comorbidity index

Diabetes

Functional disease

Hepatocellular carcinoma

Hypertension

Liver disease

Malnutrition

Metastatic Cancer

Myocardial infarction/Unstable angina/CABG

Pneumonia

Psychiatric disease

PTSD

Valvular disease

Renal Failure

Respiratory failure

Stroke

Trauma

1. Utilization:

Bed days of care (1-10 vs 0)

Cardiology visits

ER visits (>1 first year)

Mental health visits

Number of providers (>3)

Outpatient visits (>4)

Other visits (>3)

Primary care visits (>1)

Pulmonary visits

Recent Admission

1. Vital Signs:

BMI (<25)

Heart rate (>85)

Systolic & Diastolic BP

Respiratory rate (≥20)

1. Pharmacy:

ACE inhibitors/ARB

Alpha-blocker

Anti-depressants

Antiplatelet drugs

Antipsychotics

Benzodiazepine

Beta-blockers

Bumetanide/ Torsemide

Calcium channel blockers

Digoxin

Furosemide

Insulin

Lipid lowering drugs

Metformin

Metolazone

Nitrate-long acting

NSAIDS

Nebulized drugs

Opioids

Potassium sparing diuretic

PPAR-gamma agonists

Oral steroids

Thiazides

Warfarin

1. Interactions:

18 drug interaction terms
