# Supplementary material for: Validating administratively derived frailty scores for use in Veterans Health Administration emergency departments
Source: Acad Emerg Med. 2023 Mar 28;30(4):349–58. doi: 10.1111/acem.14705 (PMC10162447; doi:10.1111/acem.14705)
Supplement: Supplementary file 1 — Data S1. [file ACEM-30-349-s001.zip › acem14705-sup-0004-AppendixS4.docx]

**Appendix 4: VA-FI Data Elements**

Sourced from:

Orkaby AR, Nussbaum L, Ho YL, et al. The burden of frailty among u.s. veterans and its association with mortality, 2002-2012. Journals of Gerontology - Series A Biological Sciences and Medical Sciences 2019; 10.1093/gerona/gly232

Cheng D, Dumontier C, Yildirim C, et al. Updating and Validating the U.S. Veterans Affairs Frailty Index: Transitioning from ICD-9 to ICD-10. Journals of Gerontology - Series A Biological Sciences and Medical Sciences 2021;76(7). 10.1093/gerona/glab071

**Morbidity:**

1. Anemia

2. Atrial Fibrillation

3. Cancer (any except basal cell skin cancer)

4. Cerebrovascular disease: Stroke/TIA

5. Coronary Artery Disease: MI/CABG/PCI

6. Diabetes

7. Heart Failure (diastolic or systolic)

8. Hypertension

9. Kidney Disease: Chronic Kidney Disease, Dialysis

10. Liver Disease of Cirrhosis

11. Lung Disease: COPD or Asthma

12. Thyroid Disease

13. Osteoporosis or osteoporosis related fracture (vertebral fractures)

14. Incontinence

**Function:**

15. Arthritis (rheumatoid arthritis or osteoarthritis)

16. Use of Durable Medical Equipment

17. Fall or Fall related diagnoses: Hip fractures/ subdural hematoma/ subarachnoid hematoma

18. Fatigue

19. Gait Abnormality or difficulty walking

20. Parkinson Disease or Tremors

21. Peripheral vascular disease or Intermittent claudication

22. Muscular wasting and disuse atrophy/Cachexia/Debility

**Sensory Loss:**

23. Hearing Impairment/ Hearing Aid

24. Peripheral Neuropathy

25. Vision Comorbidity (macular degeneration, glaucoma, blindness)

26. Dementias (Alzheimer’s, Vascular, Lewy Body, Pick’s disease, Mild Cognitive Impairment)

27. Anxiety

28. Depression/Bipolar

**Other:**

29. Chronic Pain

30. Failure to thrive

31. Weight loss in the past year
